# Clinical Assessment and Diagnosis of Germline Predisposition to Hematopoietic Malignancies: The University of Chicago Experience

**DOI:** 10.3389/fped.2017.00252

**Published:** 2017-12-06

**Authors:** Ami V. Desai, Melody Perpich, Lucy A. Godley

**Affiliations:** ^1^Department of Pediatrics, Section of Hematology/Oncology and Stem Cell Transplantation, The University of Chicago, Chicago, IL, United States; ^2^Department of Medicine, Section of Hematology/Oncology, The University of Chicago, Chicago, IL, United States; ^3^Department of Human Genetics, Section of Hematology/Oncology, The University of Chicago, Chicago, IL, United States

**Keywords:** germline predisposition, inherited mutation, hereditary hematopoietic malignancy, familial mutation, genetic counseling, genetic risk assessment

## Abstract

With the increasing use of clinical genomics to guide cancer treatment and management, there is a rise in the identification of germline cancer predisposition syndromes and a critical need for patients with germline findings to be referred for surveillance and care. The University of Chicago Hematopoietic Malignancies Cancer Risk Team has established a unique approach to patient care for individuals with hereditary hematologic malignancies through close communication and coordination between our pediatric and adult programs. Dedicated program members, including physicians, nurses, genetic counselors, and clinical research assistants, screen individuals for cancer predisposition at initial diagnosis through survivorship, in addition to testing individuals with an established family history of a cancer predisposition syndrome. Sample procurement, such as a skin biopsy at the time of bone marrow aspirate/biopsy in individuals with a positive screen, has facilitated timely identification of clinical germline findings or has served as a pipeline for translational research. Our integrated translational research program has led to the identification of novel syndromes in collaboration with other investigators, which have been incorporated iteratively into our clinical pipeline. Individuals are referred for clinical assessment based on personal and family history, identification of variants in susceptibility genes *via* molecular tumor testing, and during evaluation for matched related allogeneic stem cell transplantation. Upon referral, genetic counseling incorporates education with mindfulness of the psychosocial issues surrounding germline testing at different ages. The training and role of genetic counselors continues to grow, with the discovery of new predisposition syndromes, in the age of improved molecular diagnostics and new models for service delivery, such as telemedicine. With the identification of new syndromes that may predispose individuals to hematologic malignancies, surveillance guidelines will continue to evolve and may differ between children and adults. Thus, utilizing a collaborative approach between the pediatric and adult oncology programs facilitates care within families and optimizes the diagnosis and care of individuals with cancer predisposition syndromes.

## Introduction

As health-care professionals consider the diagnosis of inherited predisposition to hematopoietic malignancies increasingly in patients with a strong personal and family history (1), coordination between pediatric and adult care teams is critical for optimal assessment of all family members across the age spectrum. Communication and coordination between pediatric and adult hematology/medical oncology groups are the hallmarks of the approach utilized by The University of Chicago Hematopoietic Malignancies Cancer Risk Team. These unique aspects of our clinical program allow a unified and fluid approach that utilizes the expertise within both programs to provide age-appropriate counseling, assessment, and testing for all members of a family. Children and adult family members can be seen together, maintaining cohesive care within a family and continuity in counseling through joint counseling. This approach has also allowed patients to receive consistent care from childhood and adolescence to adulthood, bypassing age constraints and providing dual care for the adolescent and young adult population. From a cancer risk and management perspective, description of novel germline syndromes, such as our contributions to the recently described germline mutations in *ETV6* and *DDX41* (2–4), and *SAMD9/SAMD9L* (5–8) have defined new familial predisposition syndromes, which have impacted the care and testing of both adults and their children, and further emphasize the importance of joint management.

## Identification of Individuals and Families for Germline Genetic Counseling

**Figure 1:**
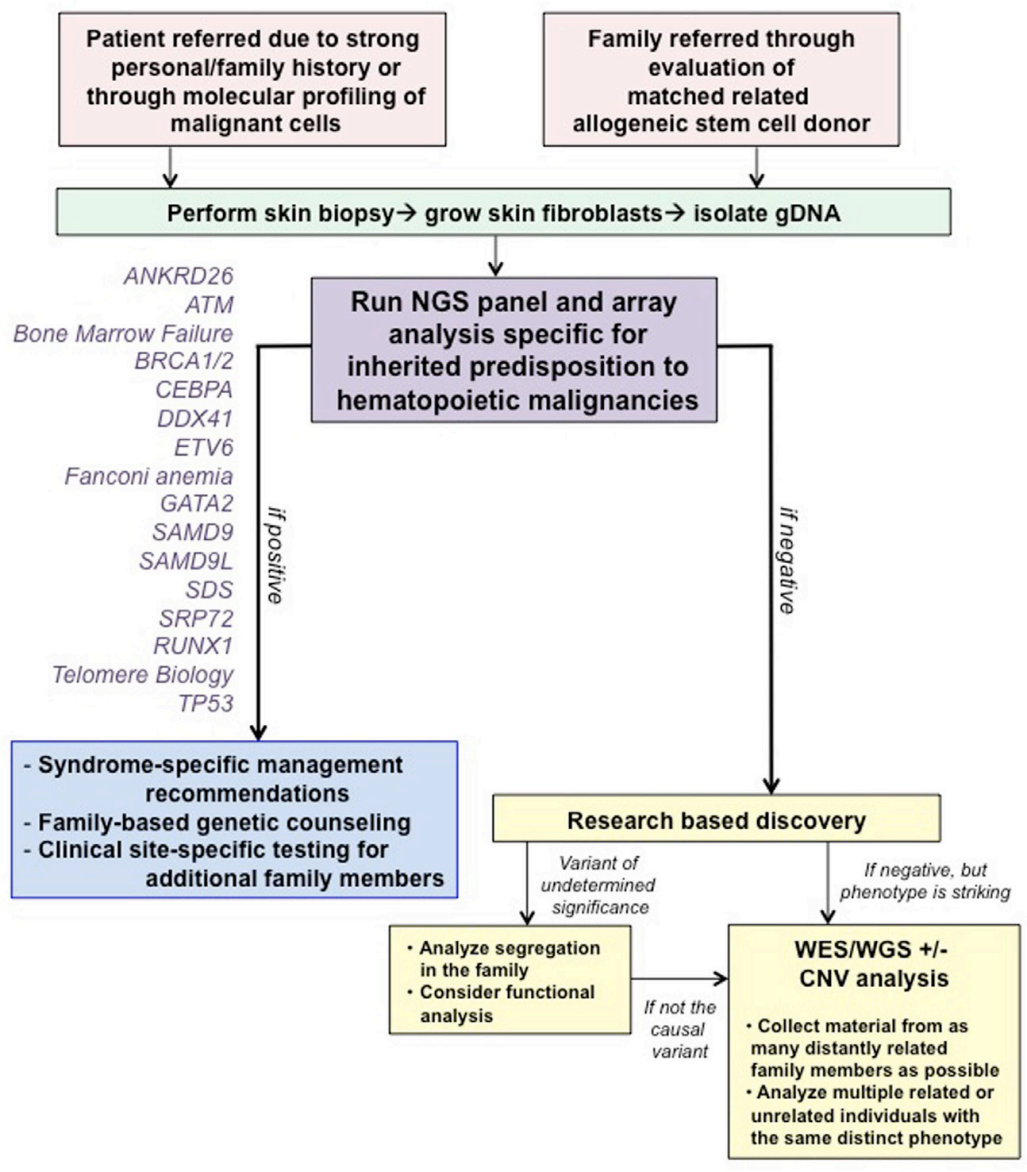
Algorithm for our clinical assessment of patients. Note that all patients undergoing clinical testing are offered participation in IRB-approved research studies to advance our knowledge about familial syndromes that predispose to hematopoietic malignancies. Abbreviations: CNV, copy number variant; WES, whole-exome sequencing; WGS, whole-genome sequencing. This figure was modified with permission from its original version, published in Ref. (1).

At our center, genetic counselors and physicians from both adult and pediatric oncology work together to identify families at risk for hereditary hematologic malignancies (HHM; Figure 1). Individuals and families are referred for genetic counseling/cancer risk assessment through various channels. First, our colleagues, both within pediatrics and adult medicine, are attuned to the existence of inherited susceptibility syndromes and have a low threshold for contacting us for an assessment. When possible, assessments are made at the time of a scheduled visit with the individual’s regular provider to minimize return clinic visits for the patient. If face-to-face discussions are not possible, then genetic counseling is performed *via* telephone or occasionally video conferencing. Second, increasingly, we are identifying variants in genes associated with a germline predisposition syndrome at the time of somatic molecular testing of hematopoietic malignancies. Although these tests were designed mainly for prognostication purposes with an assumption that they are useful for detection of somatic mutations, they cannot distinguish somatic from germline variants. Therefore, we have developed a pipeline for identification of those variants most likely to be germline, and we make every attempt to offer germline testing to those individuals. With this approach, we have identified several patients/families with germline mutations, validating the importance of this means of patient identification.

Finally, we view allogeneic stem cell transplantation as an essential time to identify families with germline predisposition syndromes necessitating germline genetic testing to prevent transplantation with an affected sibling. This is one of the only times in medicine when the relative, usually a sibling, is assessed as a patient when presenting to be the allogeneic stem cell donor. Particular findings, such as cytopenias or poor mobilization, raise suspicion of a germline syndrome and prompt a thorough evaluation (9, 10). We have identified several families with an inherited mutation in this way as well. The identification of a germline syndrome is critical at the time of allogeneic stem cell transplant, since inadvertent use of an allogeneic stem cell donor who carries a germline predisposition mutation has resulted in recipients with failure to engraft, poor graft function, posttransplant lymphoproliferative disease, and donor-derived leukemias (10–15). Moreover, those donors have also developed leukemias, raising the question as to whether the G-CSF given for mobilization has contributed to the malignancy. We have recommended the use of an unrelated donor even in cases in which a germline mutation has not been detected for families with an overwhelming history of cancer, out of concern that the family could harbor an undescribed mutation.

Identification of families with an HHM is more robust when thought of in a reciprocal manner, with the initial patient potentially identified in either the pediatric or adult setting. The first step in case identification is expanding the pedigree to include detailed information about all available relatives. It is standard of care to complete a 3-generation pedigree, but it is of increased importance to expand each pedigree further by querying about the health of all children in these families. We emphasize the importance of recognizing that the initial descriptions of germline susceptibility syndromes are likely to be incomplete, since they are by definition based on an initial small set of families, and in our experience, the phenotypes of these syndromes expands as additional individuals and families are identified (4). Therefore, it is very important to keep an open mind about which individuals/families may be at risk for an inherited syndrome. Automatic referral criteria for cancer risk genetic counseling and consideration of genetic testing can also be made based on HHM histopathologies such as juvenile myelomonocytic leukemia (neurofibromatosis 1-*NF1*; Noonan syndrome—e.g., *PTPN11, NRAS*, and *KRAS*; Noonan-like syndrome—*CBL*) (16), low hypodiploid ALL (*TP53*) (17), or myelodysplastic syndrome (MDS) diagnosed in children or young adults (*GATA2, SAMD9, SAMD9L*, and *SRP72*) (5–8, 18, 19). Individuals with suspected inherited syndromes that confer risk for additional organ manifestations beyond the bone marrow, such as Noonan syndrome or *GATA2* deficiency, should also be referred to additional specialists as appropriate (e.g., a medical geneticist for Noonan syndrome; infectious disease specialist for *GATA2* deficiency), if they have not already been evaluated by one for comprehensive management of other non-malignant conditions.

It is also important to recognize that some patients with HHM may not initially present with a suspicious family history in part due to lack of complete or accurate knowledge about relatives, cancer diagnoses, treatment, general health, or unexplained causes of death in the family. The possibility of incomplete penetrance, a *de novo* mutation or gonadal mosaicism can also provide an explanation for a single person with HHM, identified in a family, harboring a germline mutation. Germline *SAMD9* mutations are often *de novo* as are germline *GATA2* mutations arising in MDS diagnosed in young patients who lack a family history (6, 18).

In addition to families with established cancer risk, the genetic counselor in our pediatric program meets with new oncology patients who are interested in learning more about cancer risk, adolescents and young adults, and childhood cancer survivors. Patients of reproductive age, parents of patients with siblings or parents who are themselves considering having additional children, are often interested in genetic counseling and risk assessment. With an identified germline mutation, accurate testing of siblings, future pregnancies and preimplantation genetic diagnosis is possible. Having the ability to identify and provide genetic counseling for patients in various clinical settings and encompassing a wide age range has allowed for improvement in the identification and detection of high-risk hematologic malignancy predisposition families.

## Process of Obtaining Sample Procurement for Clinical Testing and Consent for Research

As we have discussed in previous reviews (1, 20, 21), we recommend clinical testing for the presence of a germline syndrome in individuals with: (i) multiple cancer diagnoses; (ii) hematopoietic malignancies diagnosed in two individuals within a 3-generation pedigree; (iii) relatives with cytopenias, hematopoietic abnormality such as macrocytosis/dysplasia, and/or poor stem cell mobilization; and (iv) relatives with other physical findings associated with a known germline predisposition syndrome (Table 1). Once genetic counseling has been performed, and an individual wants to proceed with clinical testing, an appropriate sample must be obtained. True germline material is difficult to obtain in someone with a history of a hematopoietic malignancy, since blood and bone marrow are affected tissues. Even in remission, somatic mutations associated with clonal hematopoiesis of indeterminate potential can persist in the hematopoietic compartment and be confused for a germline mutation (22, 23). This has also been reported in T-cell acute lymphoblastic leukemia, in which somatic mutations in *SMO, GLI1*, and *GLI3* were found to be present even at the time of remission, suggesting *de novo* acquisition of these mutations during early life in the hematopoietic compartment (24). In children with low hypodiploid ALL, 43.3% of *TP53* mutations were also found in non tumor hematopoietic stems cells, which may reflect an inherited origin, acquired *de novo* mutation in the germline or hematopoietic compartment (17). Other easily obtainable tissues, such as saliva and nails, can be contaminated with blood cells. Therefore, we are strict in considering only cultured skin fibroblasts as true germline tissue. Note that if the same mutation is found in any tissue from two individuals within the same family, the most likely explanation is that the variant is germline in origin, since it would be highly unlikely for two individuals to acquire the same mutation. However, since certain somatic mutations can be common, we prefer to define germline status based on variant presence in skin fibroblasts. We recognize that the culturing process itself can result in acquisition of somatic mutations/genomic rearrangements, but in our experience, this artifact results in mosaic results detectable during clinical testing. We have observed this phenomenon in less than 10% of cultures and have performed repeat skin biopsies, confirming the suspected somatic nature of these mutations in each case to date. However, because cultured skin fibroblasts provide true germline tissue as well as cells that can be grown at least to a limited degree in the research laboratory, providing a somewhat renewable source of patient material for study, we continue to prefer these cells for testing. A skin biopsy can be obtained easily at the time of a regularly scheduled bone marrow biopsy, and we have a dedicated clinical research associate who calls patients in advances and meets them at their appointment to discuss the option of performing a skin biopsy for clinical testing and for participating in research. This approach has facilitated our ability to perform skin biopsies conveniently for patients. If this is not possible, we also perform a standard 3 mm punch biopsy in clinic for patients who are not scheduled for a routine bone marrow biopsy. A drawback in using cultured skin fibroblasts as the source of germline DNA is the prolonged time it takes for the culture to grow sufficient numbers of fibroblasts, generally 3–6 weeks. Therefore, in rare cases in which the result is needed urgently for clinical management, we perform simultaneous testing of blood as the skin fibroblasts are growing. If a negative result is obtained in the blood, then there is no concern for a known predisposition syndrome. Saliva can be used as a source of germline DNA if a known predisposition syndrome exists in a particular family or in patients with long-term remissions from diseases in which cure is possible, such as Hodgkin lymphoma. At our institution, we are fortunate to be able to culture skin fibroblasts in a CLIA-certified laboratory, but this ability may not be widely available, especially to those who use outside reference laboratories.

**Table 1:** Hereditary hematopoietic malignancies screening form.

*Step 1: Draw the family pedigree using standard symbols*	

*Step 2: Utilize screening questions to guide workup* [see Table 1 of Ref. (1) for specific syndromes corresponding to letter designations]	Consider:

Do you/does anyone in your family have chronic low blood counts, including low red blood cells (anemia), low platelets (thrombocytopenia or ITP), low white blood cells (leukopenia, monocytopenia, and lymphopenia)? Has anyone required a transfusion for a low blood count?	*ANRD26, ETV6, RUNX1, GATA2, TERC/TERT*, inherited bone marrow failure syndromes, Fanconi anemia, *SAMD9*, and *SAMD9L*

Did you or did anyone in your family have thrombocytopenia and/or anemia requiring transfusions in early infancy?	*SAMD9*

Did you/does anyone in your family bleed or bruise easily? If yes, have they required transfusions for bleeding?	*ANKRD26, ETV6*, and *RUNX1*

Do you/does anyone in your family have or have had warts (genital, hands, feet, or any other site?) If yes, where and for how many years?	*GATA2*

Do you/does anyone in your family get infections easily or severe or unusual types of infections? If yes, how many infections and what type? (e.g., pneumonia, meningitis, sepsis, and fungal) and at what age(s)? Did they require hospitalization or antibiotics?	*GATA2*, inherited bone marrow failure syndromes, Fanconi anemia, and *SAMD9*

Does anyone in the family have swelling of one limb larger than the others (also known as lymphedema)? If yes, what limb and is there a known reason why that limb is swollen?	*GATA2*
Do you/does anyone in your family have deafness? If yes, at what age did it occur and is there a known reason for why that person cannot hear?	*GATA2, SRP72*, and inherited bone marrow failure syndromes

Do you/does anyone in your family have abnormal nails (e.g., misshapen or missing not due to injury)?	*TERC/TERT*, inherited bone marrow failure syndromes, and Fanconi anemia

Did you/does anyone in your family get grey hair in their 20s or earlier? Whom and at what age?	*TERC/TERT* and inherited bone marrow failure syndromes

Have you or anyone in your family had skin cancer or abnormal coloration of the skin, especially around the neck region?	*TERC/TERT*, inherited bone marrow failure syndromes, Fanconi anemia, and Lynch syndrome

Have you/anyone in your family had a specific skin problem called eczema?	*RUNX1*

Do you or does anyone in your family have lung disease, including pulmonary fibrosis, or early onset emphysema?	*TERC/TERT* and inherited bone marrow failure syndromes

Do you or does anyone in your family have a lung disease called pulmonary alveolar proteinosis?	*GATA2*

Do you/does anyone in your family have a liver disease called cirrhosis? If yes, at what age and is there a known reason why you/they have cirrhosis (for example, heavy alcohol use)?	*TERC/TERT* and inherited bone marrow failure syndromes

Have you or other family members had other types of cancer, such as head and neck cancer?	*TERC/TERT*, inherited bone marrow failure syndromes, Fanconi anemia, Li–Fraumeni syndrome (LFS), and *BRCA1/2*

Have you or other family members had other types of cancer, such as cervical or anal cancer?	*TERC/TERT*, inherited bone marrow failure syndromes, Fanconi anemia, and LFS

Have you or other family members had other types of cancer, such as early-onset breast cancer, sarcoma, or brain or colon cancers?	LFS, *BRCA1/2*, and Lynch syndrome

Did you or anyone in your family have growth restriction during both the prenatal and postnatal periods?	Inherited bone marrow failure syndromes and *SAMD9*

Do you or does anyone in your family have short stature?	Fanconi anemia and *SAMD9*

Do you or does anyone in your family have intellectual impairment?	Inherited bone marrow failure syndromes, Fanconi anemia, and *SAMD9*

Do you or does anyone in your family have adrenal insufficiency or hyperpigmentation of the skin?	Fanconi anemia, *SAMD9*

Do you or does anyone in your family have genital underdevelopment (i.e., microphallus, cryptorchidism, hypospadias, or hypoplastic ovaries)?	Fanconi anemia and *SAMD9*

Did you or did anyone in your family have delayed puberty or show limited signs of puberty?	Fanconi anemia and *SAMD9*

Are there any unexplained newborn deaths in the family?	*SAMD9*

Do you or has anyone in your family have a history of chronic diarrhea? If yes, was there colonic dilatation?	*SAMD9*

Do you or does anyone in your family have ataxia (lack of muscle coordination affecting voluntary movements such as walking)?	*SAMD9L*

*Step 3: Determine exposure history*	

Do you smoke? If yes, how many packs per day?	

Do you drink alcohol? If yes, how many drinks per day?	

Have you been exposed to pesticides? If yes, for what career, what agents, and for how many years?	

Have you been exposed to radiation and/or chemotherapy? If yes, what drugs or type of radiation were you exposed to and for what reason?	

Have you been exposed to other chemicals such as benzene? If yes, what chemicals, why, and for how long?	

Have you been exposed to endocrine disrupting chemicals (i.e., phthalate and bisphenol A) or synthetic estrogen such as diethylstilbestrol?	

*This table was modified with permission from its original version, published in Ref. (1)*.

Care should be given to which platform is used to test for germline predisposition syndromes that confer elevated risk for hematopoietic malignancies. Few tests are comprehensive for all types of gene mutations that can be causative for these syndromes, including both point mutations and gene rearrangements, most often deletions. We generally use a custom pipeline developed in the Genetic Services Laboratory at The University of Chicago,^1^ which is updated several times per year to allow addition of newly described syndromes. The University of Washington also provides the MarrowSeq panel appropriate for testing of patients with bone marrow failure-like syndromes.^2^

In our experience using a series of panels sensitive for both point mutations as well as gene rearrangements and criteria for clinical testing as described earlier, we have an overall diagnostic yield of 19% (37 of 197 patients), with a diagnostic rate of 15% for probands in the pediatric age group and 21% for adult probands (25). The diagnostic rate was highest for the dyskeratosis congenita/telomere biology and Fanconi anemia panels (25), which may reflect the more long-standing descriptions of these syndromes and hence more reliable clinical features compared with many of the more recently described HHM syndromes. Based on our and recently published experience, we believe the prevalence of pediatric leukemia predisposition is higher than previously recognized (18). There is a potential role to screen every pediatric leukemia or lymphoma patient, regardless of family history or other factors for HHM mutations. We encourage all of our patients who are undergoing clinical testing to consent to research protocols that allow the definition and characterization of novel syndromes. Thus, patients/families whose clinical testing fails to identify a known predisposition syndrome may contribute to the description of novel syndromes.

## Referral Guidelines: Best Practice 2017

A detailed past medical history, family history, and physical examination, as well as a review of key laboratory findings, are critical to identify patients with genetic predisposition for hematopoietic malignancies (Table 1). Findings such as dysmorphic features, short stature, cytopenias, immunodeficiency, specific histopathology, or toxicity out of proportion to that typically seen after chemotherapy or radiation, may indicate an underlying cancer predisposition syndrome (1, 16, 26–29). In the era of precision medicine and identification of new prognostic biomarkers, referral to our Hematopoietic Malignancies Cancer Risk Team is also driven by the use of next generation sequencing panels that are designed for use for diagnosis and prognostication, but when performed on DNA derived from affected tissue, cannot distinguish somatic from germline variants.

*TP53* is a frequently mutated gene in human cancers, although it is more frequently mutated in solid tumors compared with hematologic malignancies (30, 31). A study of both pediatric and adult cases of AML, ALL, MDS, and CLL identified alterations in *TP53* in approximately 10% of cases, with the highest frequency in ALL (31). However, *TP53* alterations were identified in 91.2% of pediatric low hypodiploid (32–39 chromosomes) ALL, with up to 40% of these patients harboring a germline mutation in *TP53* (17). Sporadic mutations in *CEBPA* (monoallelic or biallelic) have been reported 5–14% of individuals with AML (32, 33), and among those whose leukemias have biallelic mutations, germline *CEBPA* mutations were found in about 11.1% of patients (33). Germline *GATA2* mutations have been found in 15% of advanced and 7% of all primary MDS cases in children, yet germline mutations were absent in children with therapy-related MDS or acquired aplastic anemia (18). Thus, discerning the frequency of somatic versus associated germline mutations in different malignancies, as well recognition of malignancies outside of expected age ranges (i.e., MDS in the pediatric population) is essential to identifying underlying predisposition syndromes.

## Genetic Counseling

In general, the reporting of solid and hematologic malignancies occurring in families by patients to their primary health-care providers may be incomplete. Thus, to identify families at risk for HM susceptibility, it is essential to know the particular line of questions that are unique to hematologic malignancies (Table 1). Without directed questions to our patients, we may miss relevant family history information.

Standard family history questions should include the following:

1. Are there two or more relatives with a diagnosed blood cancer? We generally include relatives within two generations of the proband, both older and younger generations.
2. Are the blood cancers in the family acute or chronic? What are the specific diagnoses? Death certificates and/or medical records are helpful in confirming patient-reported data. Generally, patient-derived histories about other hematopoietic abnormalities, such as cytopenias and macrocytosis, are more difficult to elicit.
3. When mining for hereditary HM families, we also have to take a detailed family history including that of solid tumors, since many predisposition syndromes increase the likelihood for development of both solid tumors as well as hematopoietic malignancies.

## New Models of Service Delivery: Genetic Counseling Collaboration and Telegenetics

Our collaboration between the adult and pediatric oncology program has fostered identification of high-risk families, as well as communication with our families. When adult patients are identified as harboring a pathogenic mutation, placing their children at risk, it has been optimal to have a genetic counselor and oncologist available to communicate risk and management to the children of these patients in language that is tailored to their developmental ages. For our remote patients, without local genetic services, it has been necessary to accommodate and simplify the genetic counseling process by providing telemedicine services. With the use of telegenetics counseling, we can enable family participation in genetic counseling and associated genetic testing, by reducing access barriers, such as distance or shortage of local genetic counselors in a particular geographic location. We have utilized encrypted telephone conference calling services, but currently do not provide audiovisual services. To ease the telecommunication process, we have benefited from emailing a slide deck that covers information on genetics and hematologic malignancies in advance of a telecommunication/telephone counseling appointment. The information that is shared takes into consideration the developmental age and health literacy of the patient. It has been possible for us to provide genetic counseling successfully using this methodology and to obtain assent from minors before testing for a familial mutation. It is necessary to utilize HIPAA compliant technology, however, and care should be given to ensure the use of appropriate web-based platforms. We have provided encrypted teleconferencing and counseling to adult presymptomatic, mutation positive patients, and their children, with their family listening from home through use of the speaker option on their phone. Although telephone conferencing has been acceptable for our patients and was well received by the children, it is not the technological gold standard. This type of practice can be optimized with use of video education and virtual counseling with the aid of a visual, face-to-face, component. A multicenter feasibility study evaluating the use of videoconferencing to expand access to genetic services concluded that although there were a few disadvantages to real-time videoconferencing (concerns about confidentiality breach, reimbursement, technical issues), all patients reported that they were satisfied with this type of genetic service delivery model and many patients reported that there were many advantages (34). Another study provided telegenetics services to presymptomatic patients in patients’ homes, differentiating them from the prior study in which patients received video counseling at the community site and were not physically in their own homes. Similarly, patients reported satisfaction and had similar psychological outcomes when compared with patients receiving a standard in-person genetic counseling appointment (35).

At this time, we have not been able to bill for information provided *via* telegenetics communication. The ability to bill and to be reimbursed can be a requirement in many clinical practices. Another option is to consider commercial genetic counseling telegenetics services. Commercial telegenetics counseling services, however, may not have counselors with expertise in hematologic malignancies. Genetic counseling programs have not historically trained genetic counselors in the specific area of HHM and MDSs. Currently, most training in HHM occurs “on the job,” or as students, during very specific clinical rotations. Genetic counseling program curriculums would benefit from adding HMM to their standard training and subject matter.

## Psychosocial Aspects of Genetic Counseling in Pediatric Oncology

In contrast to adult cancer genetic counseling, genetic counseling in the pediatric oncology setting necessitates the need to contract with parents before consultation whether in person or remote. It is understandable that many parents are protective and concerned about upsetting their children by discussing the cancers that have occurred in the family and risk to develop cancer, particularly as it pertains to the child. Although genetic counselor providers are specifically trained to be empathic and supportive with patients and to assist families to achieve the best possible adjustment to a genetic diagnosis, there are often situations in which parents are insistent in having their young children tested for a familial mutation without their knowledge or assent. Some parents wish to inform their children after the results of their genetic test are completed and hope to accomplish the blood draw without involving them in the process. It may also be unclear how much the unaffected child knows about the cancer related deaths in a family and if they are aware, what the emotional toll has been on the child. In our experience, to help achieve alignment and consensus with such parents, it is imperative to have an *a priori* “contracting” discussion. This discussion should include the benefits of including the child in the process, the resilience, coping ability, and adaptation by many children, and how we, as providers, help children to “normalize” the testing process and the recommended cancer screening and risks after learning of a positive test result. Parents are also advised about the importance of being honest with children. The age of assent varies by state, and at our institution is 10–17 years of age. If testing is accomplished without assent from appropriate aged patients, the child may resent the parent’s decision and adversely affect the parent/child relationship in the future. In one recent publication, children surveyed who were age 10 and above indicated that they preferred to be involved in the assent process and believed that they have the right to decline testing (36). In short, without prior contracting, alignment and consensus building with parents, genetic counseling and the assent process may be suboptimal at best or unachievable at worst.

Of course in the pediatric oncology setting, many children are tested before the age of assent, without their knowledge, when they are too young to understand inheritance and the implications of a cancer diagnosis. This is more typical for families with conditions that begin cancer risk management in early childhood, such as Li–Fraumeni syndrome (LFS) or familial adenomatous polyposis. Genetic counseling, for these patients, is then typically provided at various time points as they return to our pediatric cancer risk program for management throughout the years. Genetic counseling in the pediatric, adolescent, and young adult population is a continuous process since new concerns arise as they relate to the developmental stage and maturity of the patient (i.e., reproductive risk).

## Unique Aspects of Counseling Families at Risk for Hematologic Malignancies

Opinion statements, regarding patient management once an inherited mutation has been identified, have been published by various groups (1, 28, 29, 37, 38), and consensus guidelines now exist for clinical testing (39–41). Consensus management guidelines for families with LFS have been published (42), and guidelines for clinical management for HHM in pediatrics are emerging (29).

It is difficult to standardize and simplify the patient identification process in HM families. The pedigrees we are identifying are not always straightforward, because the initial description of families with HHM is limited and based on only a few families and may not be representative of the biology of the particular disease in all families. Therefore, some families present with features that diverge from the initial syndrome description.

For example, in *DDX41* mutation families, the initial description included families with only myeloid malignancies. However, over time, we identified a family with segregation of lymphoid malignancies as well as younger age of onset (4).

In addressing the effect of variable expression and the possibility of children developing a hematologic malignancy in a family that demonstrates adult onset cancer only, we also discuss the phenomenon of anticipation, in which younger generations develop malignancy at an earlier age compared with relatives in previous generations. Anticipation is common in families with hematopoietic malignancies (43), although the molecular mechanism by which this occurs is not clear. Since the recognition of many of these syndromes is evolving and the current identification of HHM families is few in number, there is an inherent selection bias that renders it difficult to provide genetic counseling with accuracy and precision for many of these families.

## Management and Surveillance

Although published opinion papers are able to provide some guidance regarding management for family members with genetic predisposition to MDS and other hematologic malignancies (1, 28, 29, 37, 38), these recommendations are largely based on expert opinion, rather than prospective study, and therefore present limitations. Unlike LFS families for whom there are now recent and encouraging data demonstrating the benefits of cancer screening for early detection of malignancies (42, 44), there are not yet comparable studies or guidelines for HHM families to make a compelling argument for the benefit of genetic testing and cancer screening for early detection in adults let alone children. Consensus guidelines are emerging based on available information to date (29). Nonetheless, we caution readers that management and surveillance of HHM is an evolving field as new syndromes are identified. Phenotypic expression in many of these predispositions is unknown due to incomplete penetrance and variable expression. As a result, penetrance estimates and survival advantage with screening is not known or quantifiable for these families. Depending on the type of HM in a family, genetic testing can have no direct benefit. For example, the knowledge that someone is at risk for acute leukemia will not change the course of the disease or treatment once it is identified. Early detection may improve outcomes for patients with MDS or bone marrow failure since individuals can undergo allogeneic transplantation sooner, before development of acute leukemia.

Taking such situations into account, recently published guidelines suggest a CBC with platelet count and white blood cell differential at the initial visit of all patients at risk for an HHM and annually in asymptomatic patients, with more frequent CBC monitoring (ranging from every 3–6 months) at higher risk for MDS/AML. Clinical symptoms or abnormal findings in the CBC necessitate more frequent monitoring and additional testing. Annual bone marrow aspiration and biopsy is recommended for individuals at higher risk of MDS/AML, and more frequent testing should be completed for new or worsening cytopenias (29). In addition, we have experienced logistical issues with regard to management for families who do not live close to our center. We maintain flexibility to communicate with a local primary care physician if a family is comfortable having CBCs drawn locally and having results sent to us for additional review.

Regardless of the uncertainties discussed here, which we continue to share with our patients before genetic testing, the families that we currently follow have been in favor of learning as much as possible about the hereditary nature of the HM in the family for their own personal use or for the greater good of other families identified in the future. If we can identify a management plan, based on the premise of early detection, it would be worthwhile to offer genetic testing for at-risk relatives of all ages. A recent publication showed that adolescents with LFS feel that learning their mutation status was beneficial from both a management and psychological standpoint (36). Similarly, for families with an identified HHM mutation, we would expect that relatives will benefit from learning that they are at increased risk or are not at risk, if negative, for the familial HMs. Unlike LFS, the precise lifetime risk for HHM in some hereditary predisposition families is not as well understood currently and might be much lower in comparison. In cases with lower penetrance, it could be argued that genetic testing and verifying mutation status can cause unnecessary anxiety. However, many of our patients who have experienced devastating loss of beloved relatives, have expressed the desire to be involved in pioneering any improvement in the diagnosis, treatment, prevention, and cure of these conditions.

## Future Directions

Given the rapidity with which new germline predisposition syndromes are being identified, we predict that many more inherited syndromes will be described in the coming years. Therefore, maintaining a high index of suspicion and having access to genetic counseling/testing will be critical for patients and families. We also advocate participation in research studies whenever possible, so that additional syndromes can be discovered, to study the molecular progression of disease and the psychological impact of these diagnoses, and to determine if universal germline testing for patients with hematopoietic malignancies is warranted. The pediatric population presents unique opportunities and challenges, and we recommend a unified approach coordinated with adult care providers whenever possible to benefit entire family structures.

## Author Contributions

LG worked with the other authors on the outline of the review; wrote portions of the review; edited the review; and contributed to the table/figure. AD worked with the other authors on the outline of the review; wrote a substantial portion of the review; and edited the review. MP worked with the others authors on the outline of the review; wrote a substantial portion of the review; edited the review; and contributed to the table/figure.

## Conflict of Interest Statement

The authors declare that the research was conducted in the absence of any commercial or financial relationships that could be construed as a potential conflict of interest.

## Footnotes

^1^http://dnatesting.uchicago.edu.

^2^http://web.labmed.washington.edu/tests/genetics/MRW.

## References

[1] University of Chicago Hematopoietic Malignancies Cancer Risk Team. How I diagnose and manage individuals at risk for inherited myeloid malignancies. Blood (2016) 128:1800–13. 10.1182/blood-2016-05-670240 27471235 PMC5813725

[2] Zhang MY Churpek JE Keel SB Walsh T Lee MK Loeb KR Germline ETV6 mutations in familial thrombocytopenia and hematologic malignancy. Nat Genet (2015) 47:180–5. 10.1038/ng.3177 25581430 PMC4540357

[3] Polprasert C Schulze I Sekeres MA Makishima H Przychodzen B Hosono N Inherited and somatic defects in DDX41 in myeloid neoplasms. Cancer Cell (2015) 27:658–70. 10.1016/j.ccell.2015.03.017 25920683 PMC8713504

[4] Lewinsohn M Brown AL Weinel LM Phung C Rafidi G Lee MK Novel germ line DDX41 mutations define families with a lower age of MDS/AML onset and lymphoid malignancies. Blood (2016) 127:1017–23. 10.1182/blood-2015-10-676098 26712909 PMC4968341

[5] Nagamachi A Matsui H Asou H Ozaki Y Aki D Kanai A Haploinsufficiency of SAMD9L, an endosome fusion facilitator, causes myeloid malignancies in mice mimicking human diseases with monosomy 7. Cancer Cell (2013) 24:305–17. 10.1016/j.ccr.2013.08.011 24029230

[6] Narumi S Amano N Ishii T Katsumata N Muroya K Adachi M SAMD9 mutations cause a novel multisystem disorder, MIRAGE syndrome, and are associated with loss of chromosome 7. Nat Genet (2016) 48:792–7. 10.1038/ng.3569 27182967

[7] Chen DH Below JE Shimamura A Keel SB Matsushita M Wolff J Ataxia-pancytopenia syndrome is caused by missense mutations in SAMD9L. Am J Hum Genet (2016) 98:1146–58. 10.1016/j.ajhg.2016.04.009 27259050 PMC4908176

[8] Schwartz JR Wang S Ma J Lamprecht T Walsh M Song G Germline SAMD9 mutation in siblings with monosomy 7 and myelodysplastic syndrome. Leukemia (2017) 31:1827–30. 10.1038/leu.2017.142 28487541 PMC5540771

[9] Churpek JE Nickels E Marquez R Rojek K Liu B Lorenz R Identifying familial myelodysplastic/acute leukemia predisposition syndromes through hematopoietic stem cell transplantation donors with thrombocytopenia. Blood (2012) 120:5247–9. 10.1182/blood-2012-09-457945 23258901

[10] Rojek K Nickels E Neistadt B Marquez R Wickrema A Artz A Identifying inherited and acquired genetic factors involved in poor stem cell mobilization and donor-derived malignancy. Biol Blood Marrow Transplant (2016) 22:2100–3. 10.1016/j.bbmt.2016.08.002 27497531 PMC5592729

[11] Buijs A Poddighe P van Wijk R van Solinge W Borst E Verdonck L A novel CBFA2 single-nucleotide mutation in familial platelet disorder with propensity to develop myeloid malignancies. Blood (2001) 98:2856–8. 10.1182/blood.V98.9.2856 11675361

[12] Fogarty PF Yamaguchi H Wiestner A Baerlocher GM Sloand E Zeng WS Late presentation of dyskeratosis congenita as apparently acquired aplastic anaemia due to mutations in telomerase RNA. Lancet (2003) 362:1628–30. 10.1016/S0140-6736(03)14797-6 14630445

[13] Owen CJ Toze CL Koochin A Forrest DL Smith CA Stevens JM Five new pedigrees with inherited RUNX1 mutations causing familial platelet disorder with propensity to myeloid malignancy. Blood (2008) 112:4639–45. 10.1182/blood-2008-05-156745 18723428

[14] Xiao H Shi J Luo Y Tan Y He J Xie W First report of multiple CEBPA mutations contributing to donor origin of leukemia relapse after allogeneic hematopoietic stem cell transplantation. Blood (2011) 117:5257–60. 10.1182/blood-2010-12-326322 21403128

[15] Liew E Owen C. Familial myelodysplastic syndromes: a review of the literature. Haematologica (2011) 96:1536–42. 10.3324/haematol.2011.043422 21606161 PMC3186316

[16] Stieglitz E Loh ML. Genetic predispositions to childhood leukemia. Ther Adv Hematol (2013) 4:270–90. 10.1177/2040620713498161 23926459 PMC3734905

[17] Holmfeldt L Wei L Diaz-Flores E Walsh M Zhang J Ding L The genomic landscape of hypodiploid acute lymphoblastic leukemia. Nat Genet (2013) 45:242–52. 10.1038/ng.2532 23334668 PMC3919793

[18] Wlodarski MW Hirabayashi S Pastor V Starý J Hasle H Masetti R Prevalence, clinical characteristics, and prognosis of GATA2-related myelodysplastic syndromes in children and adolescents. Blood (2016) 127:1387–97. 10.1182/blood-2015-09-669937 26702063

[19] Babushok DV Bessler M Olson TS. Genetic predisposition to myelodysplastic syndrome and acute myeloid leukemia in children and young adults. Leuk Lymphoma (2016) 57:520–36. 10.3109/10428194.2015.1115041 26693794 PMC4798888

[20] Feurstein S Drazer MW Godley LA Genetic predisposition to leukemia and other hematologic malignancies. Semin Oncol (2016) 43:598–608. 10.1053/j.seminoncol.2016.10.003 27899193

[21] Brown AL Churpek JE Malcovati L Dohner H Godley LA. Recognition of familial myeloid neoplasia in adults. Semin Hematol (2017) 54:60–8. 10.1053/j.seminhematol.2016.11.003 28637618

[22] Genovese G Kähler AK Handsaker RE Lindberg J Rose SA Bakhoum SF Clonal hematopoiesis and blood-cancer risk inferred from blood DNA sequence. N Engl J Med (2014) 371:2477–87. 10.1056/NEJMoa1409405 25426838 PMC4290021

[23] Jaiswal S Fontanillas P Flannick J Manning A Grauman PV Mar BG Age-related clonal hematopoiesis associated with adverse outcomes. N Engl J Med (2014) 371:2488–98. 10.1056/NEJMoa1408617 25426837 PMC4306669

[24] Dagklis A Pauwels D Lahortiga I Geerdens E Bittoun E Cauwelier B Hedgehog pathway mutations in T-cell acute lymphoblastic leukemia. Haematologica (2015) 100:e102–5. 10.3324/haematol.2014.119248 25527561 PMC4349289

[25] Guidugli L Johnson AK Alkorta-Aranburu G Nelakuditi V Arndt K Churpek JE Clinical utility of gene panel-based testing for hereditary myelodysplastic syndrome/acute leukemia predisposition syndromes. Leukemia (2017) 31:1226–9. 10.1038/leu.2017.28 28104920 PMC5420790

[26] Seif AE. Pediatric leukemia predisposition syndromes: clues to understanding leukemogenesis. Cancer Genet (2011) 204:227–44. 10.1016/j.cancergen.2011.04.005 21665176

[27] West AH Godley LA Churpek JE. Familial myelodysplastic syndrome/acute leukemia syndromes: a review and utility for translational investigations. Ann N Y Acad Sci (2014) 1310:111–8. 10.1111/nyas.12346 24467820 PMC3961519

[28] Furutani E Shimamura A. Germline genetic predisposition to hematologic malignancy. J Clin Oncol (2017) 35:1018–28. 10.1200/JCO.2016.70.8644 28297620 PMC5559882

[29] Porter CC Druley TE Erez A Kuiper RP Onel K Schiffman JD Recommendations for surveillance for children with leukemia-predisposing conditions. Clin Cancer Res (2017) 23:e14–22. 10.1158/1078-0432.CCR-17-0428 28572263

[30] Preudhomme C Fenaux P The clinical significance of mutations of the P53 tumour suppressor gene in haematological malignancies. Br J Haematol (1997) 98:502–11. 10.1046/j.1365-2141.1997.2403057.x 9332302

[31] Stengel A Kern W Haferlach T Meggendorfer M Fasan A Haferlach C. The impact of TP53 mutations and TP53 deletions on survival varies between AML, ALL, MDS and CLL: an analysis of 3307 cases. Leukemia (2017) 31:705–11. 10.1038/leu.2016.263 27680515

[32] Pabst T Mueller BU. Transcriptional dysregulation during myeloid transformation in AML. Oncogene (2007) 26:6829–37. 10.1038/sj.onc.1210765 17934489

[33] Pabst T Eyholzer M Haefliger S Schardt J Mueller BU. Somatic CEBPA mutations are a frequent second event in families with germline CEBPA mutations and familial acute myeloid leukemia. J Clin Oncol (2008) 26:5088–93. 10.1200/JCO.2008.16.5563 18768433

[34] Bradbury A Patrick-Miller L Harris D Stevens E Egleston B Smith K Utilizing remote real-time videoconferencing to expand access to cancer genetic services in community practices: a multicenter feasibility study. J Med Internet Res (2016) 18:e23. 10.2196/jmir.4564 26831751 PMC4754531

[35] Otten E Birnie E Ranchor AV van Langen IM. Telegenetics use in presymptomatic genetic counselling: patient evaluations on satisfaction and quality of care. Eur J Hum Genet (2016) 24:513–20. 10.1038/ejhg.2015.164 26173963 PMC4929881

[36] Alderfer MA Lindell RB Viadro CI Zelley K Valdez J Mandrell B Should genetic testing be offered for children? The perspectives of adolescents and emerging adults in families with Li-Fraumeni syndrome. J Genet Couns (2017). 10.1007/s10897-017-0091-x 28303452

[37] Churpek JE Lorenz R Nedumgottil S Onel K Olopade OI Sorrell A Proposal for the clinical detection and management of patients and their family members with familial myelodysplastic syndrome/acute leukemia predisposition syndromes. Leuk Lymphoma (2013) 54:28–35. 10.3109/10428194.2012.701738 22691122

[38] Godley LA Shimamura A. Genetic predisposition to hematologic malignancies: management and surveillance. Blood (2017) 130:424–32. 10.1182/blood-2017-02-735290 28600339 PMC5533201

[39] Arber DA Orazi A Hasserjian R Thiele J Borowitz MJ Le Beau MM The 2016 revision to the World Health Organization classification of myeloid neoplasms and acute leukemia. Blood (2016) 127:2391–405. 10.1182/blood-2016-03-643544 27069254

[40] Dohner H Estey EH Amadori S Appelbaum FR Büchner T Burnett AK Diagnosis and management of acute myeloid leukemia in adults: 2016 recommendations from an International Expert Panel, on behalf of the European LeukemiaNet. Blood (2017) 129:424–47. 10.1182/blood-2016-08-733196 19880497

[41] Greenberg PL Stone RM Al-Kali A Barta SK Bejar R Bennett JM Myelodysplastic syndromes, version 2.2017, NCCN clinical practice guidelines in oncology. J Natl Compr Canc Netw (2017) 15:60–87. 10.6004/jnccn.2017.0007 28040720

[42] Villani A Shore A Wasserman JD Stephens D Kim RH Druker H Biochemical and imaging surveillance in germline TP53 mutation carriers with Li-Fraumeni syndrome: 11 year follow-up of a prospective observational study. Lancet Oncol (2016) 17:1295–305. 10.1016/S1470-2045(16)30249-2 27501770

[43] Tegg EM Thomson RJ Stankovich JM Banks A Marsden KA Lowenthal RM Anticipation in familial hematologic malignancies. Blood (2011) 117:1308–10. 10.1182/blood-2010-07-296475 21115977

[44] Villani A Tabori U Schiffman J Shlien A Beyene J Druker H Biochemical and imaging surveillance in germline TP53 mutation carriers with Li-Fraumeni syndrome: a prospective observational study. Lancet Oncol (2011) 12:559–67. 10.1016/S1470-2045(11)70119-X 21601526

